# Status Epilepticus: Epidemiology and Public Health Needs

**DOI:** 10.3390/jcm5080071

**Published:** 2016-08-16

**Authors:** Sebastián Sánchez, Fred Rincon

**Affiliations:** 1 Grupo de Neurociencias de Antioquia, University of Antioquia, Medellin 00523, Colombia; sebassanchez05@gmail.com; 2 Department of Neurosurgery, Division of Critical Care and Neuro-Trauma, Thomas Jefferson University, Philadelphia, PA 19106, USA

**Keywords:** epilepsy, epidemiology, public health, incidence, prevalence, etiology, prognosis, population, mortality, costs

## Abstract

Status epilepticus (SE) is defined as a continuous clinical and/or electrographic seizure activity lasting five minutes or more or recurrent seizure activity without return to baseline. There is a paucity of epidemiological studies of SE, as most research is derived from small population studies. The overall incidence of SE is 9.9 to 41 per 100,000/year, with peaks in children and the elderly and with febrile seizures and strokes as its main etiologies. The etiology is the major determinant of mortality. Governments and the academic community should predominantly focus on the primary prevention of etiologies linked to SE, as these are the most important risk factors for its development. This review describes the incidence, prevalence, etiology, risk factors, outcomes and costs of SE and aims to identify future research and public health needs.

## 1. Introduction

Status epilepticus (SE) is a life-threatening neurological emergency that requires prompt diagnosis and treatment. The original definitions of SE have evolved into a more narrow and sensitive description that reflects the seriousness of this disease and the aggressiveness with which it ought to be approached. The work done by Meldrum suggests that 82 min or more of ongoing seizure activity in baboons can cause irreversible neuronal injury [1]. This is what led to the original 30 min definition; but some clinicians argue that seizures not treated within the first five minutes already carry a bad prognosis for the patient. The International League Against Epilepsy (ILAE) recently proposed a new definition for SE based on the knowledge of pathophysiology and the need for clinical treatment. SE is a condition resulting from the failure of the mechanisms responsible for seizure termination or initiation of the mechanisms that lead to abnormally prolonged seizure (>30 min) [2,3,4,5]. Because of its association with secondary neuronal injury, the 5-min time-frame is accepted as the guide for the initiation of emergency treatment, especially in comatose or stuporous patients in whom seizure activity is suspected [6].

SE itself is a condition for which data on the incidence, etiology, risk factors and outcomes are thus required for the decision-making process and for the allocation of resources by institutions and government agencies. These resources need to be used in the development of strategies that help improve prevention, diagnosis, attention and SE-related morbimortality. Much of the available empirical data on SE is derived from small population-based studies, predominantly from the United States (U.S.). Studies on the epidemiology of SE in other countries, and especially in the developing world, are scarce. The reasons for this include under ascertainment of SE cases, particularly outside of academic referral centers; lack of appropriate funding for research; difficulty in defining populations at risk; scarcity of medical centers with expertise; and not having a clear definition of SE [7,8]. There’s also emerging evidence that among epileptic disorders, SE is associated with prolonged intensive care treatment and high indirect costs because of its unfavorable outcome of premature death or loss of productivity, and it is unknown if the introduction of newer antiepileptic drugs (AEDs) causes an increase in direct costs or might even be cost-effective in preventing prolonged hospital admissions [9].

We performed a non-systematic review of the literature in relation to the epidemiology of SE, centered on the incidence, prevalence, etiology/risk factors, mortality, prognosis and costs. We aim to identify where we are currently and the critical areas where we need to do additional work to satisfy public health needs regarding SE.

## 2. Methods

A computer-based literature search of PubMed was performed using the following Medical subject headings (MeSH) terms and strategies:

status epilepticus, filtered by observational studies, reviews and systematic reviews.
status epilepticus and public health.
status epilepticus and epidemiology.
status epilepticus and incidence.
status epilepticus and prevalence.
status epilepticus and etiology.
status epilepticus and population.
status epilepticus and mortality.
status epilepticus and prognosis.
status epilepticus and costs.

The search was not limited by language, time period or age. The reference list of articles identified in each of the papers was examined to identify additional studies. No gray literature search was performed. All prospective or retrospective population-based studies and reviews where the main focus was the epidemiology, incidence, prevalence, etiology, morbimortality, prognosis, outcomes and/or cost were included. If there were any doubts, full-text was reviewed by both authors to ascertain the article’s suitability. Studies of all age groups were included. Case series, case reports, clinical trials, conference proceedings, abstracts or reviews of SE not involving any of the above terms or where the main focus was diagnosis, pathophysiology or treatment were excluded.

## 3. Results

**Figure 1:**
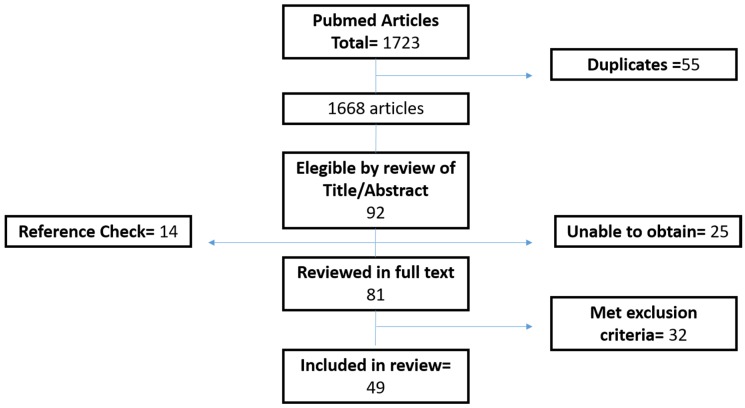
Study flowchart showing the process for the selection of articles to be reviewed.

A total of 1723 articles were found by the search strategy. Fifty-five duplicates were excluded. Based on title and abstract, 92 articles met our pre-defined inclusion criteria, and after checking the reference list of each paper, 14 new articles were added. Twenty-five articles could not be obtained for full-text review. Eighty-one articles were reviewed in full-text. Thirty-two of these met the exclusion criteria. Finally, 49 papers were used for the review (Figure 1).

### 3.1. Demographics

**Figure 2:**
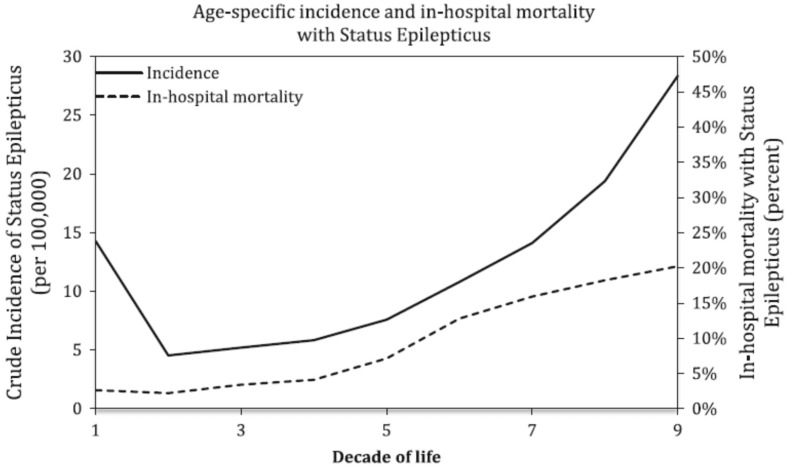
Age-specific crude incidence and in-hospital mortality with status epilepticus. Taken and adapted from Dham et al. [8].

Using data from the National Health Discharge Survey (NHDS) from 1979–2010, Dham et al. found [10] that a total of 760,117 discharges with a diagnosis of SE were identified among over one billion hospitalizations, which accounted for 0.07% of all hospital admissions in the U.S. over the 32-year period. The overall gender distribution was fairly even between males and females, with 50.3% and 49.7%, respectively. The majority of the discharges were whites, accounting for 64% of the cumulative sample. A higher incidence of SE is found in males, with a mean annual relative risk (RR) of 1.1 (95% C.I. 1.1–1.2, *p* < 0.0001). The incidence of SE had a bimodal distribution, with high incidence in the first decade of life (14.3/100,000/year) and after 60 years (28.4/100,000/year) (Figure 2).

Prospective population-based studies like the ones conducted in Rochester, Minnesota, and Richmond, Virginia, show a similar bimodal distribution of SE with an annual incidence rate of SE in children under one year of age and the elderly above 60 years age of 156/100,000/year and 86/100,000/year, respectively, and a recurrence rate of approximately 13% [11,12]. Changes in the demographic profile in developed and developing countries have revealed the fastest growth to be in the older age group; therefore, SE is likely to become a common problem and an important health issue in years to come [13]. In the Asian continent, a not-so-different demographic profile is found, with a fairly equal sex distribution with 53%–57% males, a male-to-female ratio of 1.35:1 and an incidence of the first SE episode of 42/100,000/year [14,15,16]. Meanwhile, countries like Italy, France and Switzerland have an inverse male-to-female ratio [17,18,19,20]. According to the study by Chin et al., the incidence of SE in children 15 or younger in the U.K. is 14.5/100,000/year, being the highest for those aged one year or less with an incidence of 51/100,000/year [21]. Central and South American countries mirror the results of North America, with a male preponderance in SE and a peak in incidence in the first year of life [22,23]. Some investigators have shown that countries like Honduras have an incidence of epilepsy that surpasses that of industrialized nations with an incidence of 104/100,000/year, with many of them due to possibly preventable causes [8]. An African study conducted by Amare et al reports a male-to-female ratio of SE of 1.5:1 [24].

### 3.2. Risk Factors, Etiology and Comorbidities

**Figure 3:**
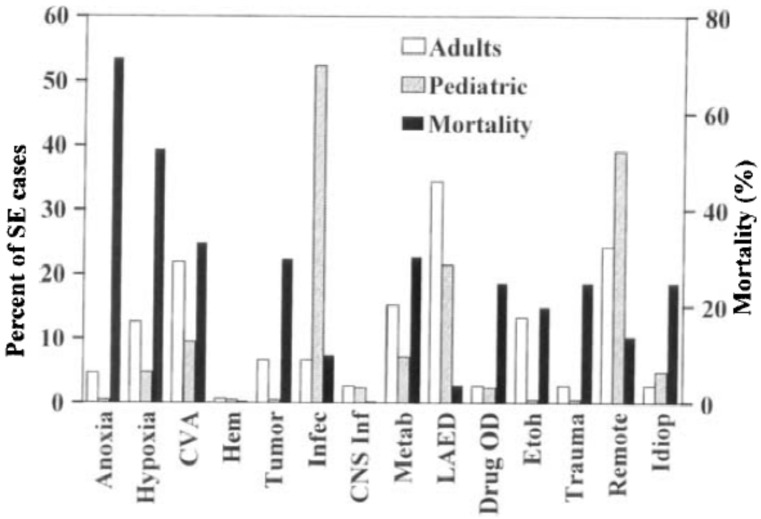
Etiologies of status epilepticus (SE) for adults and pediatric patients and mortality for adult etiologies. CVA, cerebrovascular accidents Taken from: DeLorenzo et al. [11]. Hem, hemorrhage; Infec, systemic infections with fever; CNS Infec, infections of the central nervous system; Metab, metabolic; LAED, low antiepileptic drug levels; Drug OD, drug overdose; ETOH, alcohol-related; Idiop, idiopathic.

Risk factors for SE are strongly linked to its etiology, and there is a significant variation in these when analyzed by age group. Two population-based studies report similar findings, describing infection with fever not involving the central nervous system (CNS) as the major etiology of SE in children, accounting for 52% of the cases, followed by remote CNS insult (39%) and low anticonvulsant drug levels (21%); meanwhile, three major etiologies were observed in adults: low anticonvulsant drugs (34%), remote symptomatic epilepsy (24%) and stroke (22%) [11,25] (Figure 3). The most important cause of SE in the very elderly is cerebrovascular accident (CVA), as depicted by Canouï in a case-control study with 70 patients over 70 years who suffered SE compared against non-SE matched controls. The study found that stroke (O.R. 7.9 95% C.I. 3.3–19.15), along with trauma, acute cardiac, respiratory or hepatic decompensation, epilepsy and dysnatremia were factors significantly associated with SE [19]. In a recent study of a large administrative database, Urtecho et al. found that risk factors for SE in patients with severe sepsis and septic shock were younger age, female gender, black race, the presence of metabolic derangements and renal and respiratory dysfunction. Despite a significant reduction in sepsis-related mortality, this study showed that the presence of SE has more than doubled the in-hospital mortality in severe sepsis and septic shock [26].

For the Chinese adult population, the most common etiology was cerebrovascular disease (27%), followed by idiopathic (18%) and metabolic derangement (16%) and non-compliance with AEDs (14%) [14], while for 120 children <15 years of age between 2003 and 2005 in Japan, febrile SE accounted for 49.3% of cases, followed by acute symptomatic cases (17.5%) [16]. For the European population aged 20 or older, acute symptomatic SE ranges from 34%–60%, with a cerebrovascular pathology as the most common cause [17,18,20]. In the study by Chin et al. [21], 266 children were enrolled for the ascertainment of SE. Ninety-eight children (56%) were previously neurologically healthy individuals. Acute symptomatic SE occurred in 30 cases (17%). Most children with acute symptomatic SE (17%) had either a metabolic derangement (electrolyte imbalance, hypoglycemia, hypocalcemia or hypomagnesemia) or an acute CNS infection (12% bacterial meningitis versus, 8% viral CNS infection). Remote symptomatic and cryptogenic accounted for 16% and 12% of SE cases, respectively.

For the developing world, infectious diseases seem to play an important role as an etiologic factor. A prospective study in Sao Paolo, Brazil, recollected data from 102 patients with SE admitted to a local hospital emergency department. Patients were subdivided into two groups: A, consisting of epileptic patients, and B, individuals with no previous history of epilepsy. In Group A, the main causes of SE were non-compliance with AEDs (31.8%) and undetermined etiology (39%) (*p* < 0.05). In Group B, three etiologies predominated: CNS infection (26.6%), stroke (24.4%) and metabolic disturbances (17.7%) (*p* < 0.05) [21]. Other studies from Central and South America identify irregular use of AEDs, sleep deprivation, heavy alcohol use and illicit drug use as risk factors for the development of SE and traumatic brain injury (TBI), idiopathic epilepsy and neurocysticercosis acting as important etiologies [8,23]. Sadarangani et al., in their cohort study of Kenyan children, found that 71% of SE cases had an infectious cause, 53% attributed to malaria [27]. Likewise, Amare et al. described CNS infections as the primary source of SE episodes in 119 Ethiopian patients aged 13 or older. The specific etiologies for these CNS infections were cerebral toxoplasmosis and meningitis in 18 and 14 patients, respectively [24].

There are other risk factors non-related to etiology for patients with SE. A population-based twin study reported a high pattern of SE concordance between monozygotic twins compared to dizygotic twins, linking familial predisposition and possible genetics factors to the risk of developing SE [28].

### 3.3. Acute Complications and Mortality

Although SE is more common in children than in adults, the short- and long-term mortality associated with SE is significantly lower in children [29,30], but some question the extent to which age per se renders an individual more or less susceptible to adverse outcomes [31]. Several authors show that the likelihood of death and poor outcomes increases with age after adjusting for other covariates, including age, sex, possible etiology and potential medical complications, especially for those older than 80 years [32,33]. However, most agree that the main determinant of adverse outcomes is etiology, which varies considerably depending on the age group (Table 1). Febrile SE is an important etiology of SE in children, but it was noted to have a low mortality (1.6%) [34]; and most deaths during hospitalization occur in children with acute or remote symptomatic causes [35]. In the adult population, SE was associated with a mortality of 26% that could rise up to 50% in those aged >80 years. CVA plays a major role in mortality, contributing to a mortality rate of approximately 40% [36], while cardiovascular, CNS infections, TBI, systemic metabolic derangements and progressive symptomatic etiologies have at least a 30% mortality rate [13,37]. SE cases associated with low AED levels and alcohol abuse have relatively good prognoses, with reported mortality less than 10% [38].

In a large U.S. cohort, respiratory failure necessitating endotracheal intubation was the leading complication of generalized convulsive status epilepticus (GCSE). Infectious complications, even though less prevalent, were associated with higher mortality (38.3%) [10].

**Figure 4:**
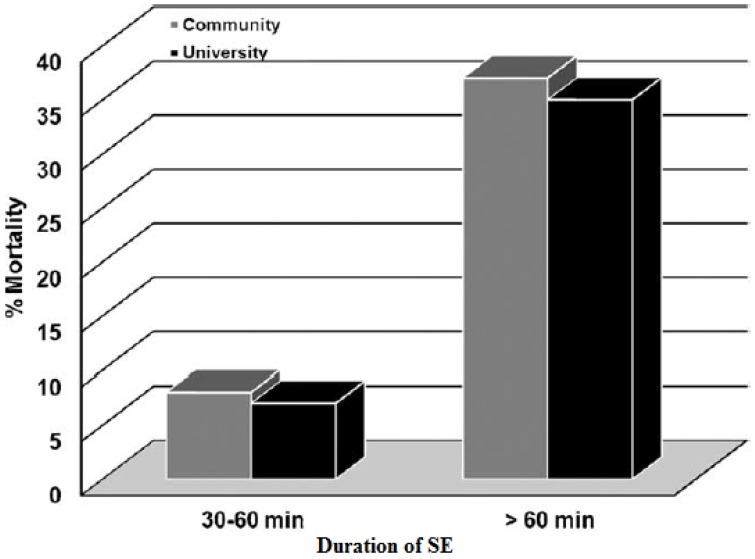
Seizure duration and outcome for private practice community and university hospitals Taken and adapted from: DeLorenzo [39].

Other important factors associated with long-term mortality are status type and duration of SE. The long-term mortality rate at 10 years was higher for myoclonic SE, for SE lasting more than 60 min, and for acute symptomatic SE [12]. Two different prospective population-based studies showed how patients with seizures lasting more than 60 min had a higher mortality rate than those who seized 30–60 min, after adjusting for other variates, like age and etiology [39,40] (Figure 4). Directly related to the duration of SE is the delay in treatment. Hui et al. [12] defined a delay in treatment as the initiation of AEDs 30 min after the onset of SE (one hour later, based on the 30 minutes definition of SE). This delay in treatment was associated with poorer outcome in multivariate analysis (O.R. 3.51, *p* = 0.04).

The question of whether or not seizures, and particularly SE, are risk factors for cognitive dysfunction has been debated for many years. In children, morbidity following SE remains low in the absence of acute symptomatic or progressive cause, with most high-quality studies reporting less than 15% developing new neurological deficits as a result of SE [41]. Epilepsy is the most problematic long-term complication of SE; however, as in other aspects of this disease, it is difficult to differentiate the risk of ongoing epilepsy following SE itself from the underlying condition. Hesdorffer et al. looked at this issue in their 10-year follow-up study. After multivariate analysis controlling for etiology, age and sex, acute symptomatic SE was associated with a 3.3-fold increase in the risk of subsequent epilepsy after a first unprovoked seizure compared to those with acute symptomatic seizures only [42]. Other authors have reviewed the literature for the relationship between neurological impairment, cognitive sequelae and SE. Again, etiology appears to be the main determinant of this outcome. Of children with acute symptomatic SE, new neurological dysfunction occurs in about 20% of cases. However, in the absence of an acute or progressive neurological disorder, new neurological deficits occur in less than 10% of cases of childhood convulsive SE [30]. Ferro shows how epileptic children who suffer SE will have more comorbid cognitive problems and worse quality of life (QOL) as measured by the Quality of life in childhood epilepsy questionnaire (QOLCE) after a 24-month follow-up [43]. There are less data about functional outcomes in adults following SE. In their retrospective study, Claassen et al. identified 10 variables associated with functional disability in univariate analysis. Of these, acute symptomatic seizure (odds ratio (O.R.) 3.9, *p* < 0.05; C.I. 1.0–14.7) and lengthy hospitalization (O.R. 1.04, *p* > 0.01; C.I. 1.0–1.1) were statistically significant [44].

### 3.4. Costs

Cost of illness (COI) studies have been carried out for SE. Penberty et al. analyzed prospectively 192 patients in all age groups and retrieved data on the direct costs for reimbursements due to inpatient admissions. The estimated direct costs summed up to US$ 18,834 per admission. The adult age group (17–45 years) was responsible for the highest amount, with a median cost of US$14,689. Increased median costs were associated with admissions due to an acute CNS etiology, in comparison to non-acute CNS etiology, with US$16,919 and $6669, respectively. Projected annual direct costs due to inpatient admissions for SE were US$4 billion [45]. Another COI study performed in Marburg-Biedenkopf, Germany, with 145 patients admitted to the hospital for epilepsy or SE determined that the cost of inpatient treatment of SE was significantly higher than the in-patient treatment of patients with newly-diagnosed or established epilepsy (€8,347 vs. €1,998 and €3,475). Treatment of SE associated with an acute illness and no previous diagnosis of epilepsy showed a tendency to produce higher costs in general (€11,934). Seizure-related injuries resulted in a mean hospitalization cost of €3399 per patient/admission and a total annual hospital inpatient cost of €1038. These data, extrapolated to the approximately 82 million inhabitants in Germany, results in SE-related hospital costs of €83 million per year [46]. Multivariate analyses of the previous studies demonstrate that age and etiology act as significant independent predictors for higher costs [9].

## 4. Discussion

In the pediatric population, the most frequent etiologies for the development of SE are infections, brain injury and epilepsy, while in the adult population, major etiologies include CVA, withdrawal or changes in AEDs, as well as remote symptomatic epilepsy. Although most experts agree that the underlying etiology is mainly responsible for the mortality related to SE, some studies have shown that age by itself can render a particular individual more susceptible to adverse outcomes after SE [32,33]. Studies carried out in different populations around the globe agree that SE has a bimodal age distribution, occurring mostly in children <1 year of age and adults aged older than 60 years [10,14,15,17,20,29]. Even though SE is more common in children than in adults, the risk of death associated with SE is significantly lower in children [30]. According to the World Health Organization (WHO), in almost every country, the proportion of people aged over 60 years is growing faster than any other age group, as a result of both longer life expectancy and declining fertility rates [47]. Therefore, with the anticipated growth of this population, SE is likely to become a more common problem and an important public health issue.

Preventive strategies to decrease the burden of TBI and stroke will likely influence the incidence of SE in the adult population. It is important to note however that other factors can influence the positive outcomes of these strategies. A study conducted by Morgenstern between 1990 and 2010, showed a decrease of 13% in the incidence of ischemic stroke in high-income countries, with no significant change seen in low- or middle-income countries [48]. For both the adult and pediatric populations, especially those with an underlying neurological defect, several studies have shown that withdrawal or change in AEDs is associated with the development of an SE crisis [25,38]. Even though withdrawal or change in AEDs is one of the main trigger factors for SE, some studies suggest that up to 48% of SE cases in the setting of known epilepsy occurred despite optimal AED levels [49]. Public health initiatives to decrease the burden of cerebrovascular disease, the careful review of AEDs and their therapeutic levels and the timely treatment of any underlying disease are important etiology-focused strategies that may help decrease the burden of SE.

Under-developed and developing countries have a higher incidence of SE, which surpasses that of industrialized nations [50]. Infectious causes are the main etiology of SE in these regions. Malaria is a common cause of childhood infection in Sub-Saharan African and South American countries and also has a higher likelihood of CNS complications when compared to malaria-infected adults [51,52]. Other population-based studies estimate that CNS infections account for 19% of cases of SE, with some of these patients having meningitis or encephalitis [25]. Infectious diseases being a preventable and reversible etiology for SE, efforts should be focused on the development of anti-malaria and vector-controlling campaigns, vaccination strategies for meningoencephalitis-prone microorganisms and efficacious access to health services for opportune diagnosis and treatment. These strategies may have an important effect on the reduction of SE, since infections represent a major cause of SE in high-risk populations, like children and the elderly. Nevertheless, significant socio-political obstacles can present along the way for these strategies to come true, and in those cases where SE has already been established, several countries lack a high-complexity unit that focuses on the treatment of SE [18,27].

Finally, even though there is a paucity of health economic data describing the costs associated with the acute management of SE or its long-term sequelae, data from the studies performed by Penberthy and Strzelczyk estimate SE-related direct costs of about US$4 billion and €83 million per year, respectively. In comparison to other acute health emergencies, like myocardial infarction, congestive heart failure or intracerebral hemorrhage, the reimbursement for SE is 60% higher. Potential elements for increased costs in SE are prolonged admission and use of newer AEDs, which can be more expensive, but do not necessarily have more efficacy in treating the condition [45,46]. It is clear that SE is a very expensive disease, and focusing on prevention could have significant benefits for healthcare associated costs. This is particularly important for low-income countries, where the scarcity of resources makes SE a disease that does not get the treatment it needs, a treatment that is often of high complexity.

In conclusion, SE is a neurological emergency that requires prompt treatment in order to improve the chances for a successful outcome. The epidemiology of SE has remained unchanged for several years, and public health interventions at the moment are limited. Governments and the academic community should focus mainly on the primary prevention of SE: highly prevalent etiologies among high-risk age groups should have especial attention paid to them (CNS infections in children, TBI in young patients, CVA in the elderly); research around the pathophysiology and triggers of SE; and the development of low-cost effective disease-modifying AEDs. The development of only one of these strategies may be insufficient, but a multi-modal approach would help reduce the health-related and socio-economic burden of SE and its sequela.

## Figures and Tables

**Table 1:** Frequency and prognosis of status epilepticus of different causes.

Cause	Incidence	Population	Mortality
Stroke	20%	Elderly	20%–40%
Alcohol abuse	8.1%–25%	Adults	0%–10%
Drug abuse	2%–14%	Adults	20%
AED reduction or withdrawal or low AED levels	34%	Adults	
Severe acute anoxia/hypoxia	8%–13%	Adults	60%–80%
CNS infection ^1^	1%–12%	Children	30%
Brain tumors	2%–15%	Adults	0%–20%
Trauma	0%–10%	Adults	11%–25%
Cryptogenic	5%		Variable

Adapted from Neligan et al. [38]; ^1^ greater in developing countries [30]; AED, antiepileptic drugs; CNS, central nervous system.

## References

[1] Meldrum B.S., Horton R.W. (1973). Physiology of status epilepticus in primates. Arch. Neurol..

[2] Lowestein D.H. (1999). Status epilepticus: An overview of the clinical problem. Epilepsia.

[3] Lowestein D.H., Alldredge B.K. (1998). Status Epilepticus. N. Engl. J. Med..

[4] Lowestein D.H., Bleck T., Macdonald R.L. (1999). It’s time to revise the definition of Status Epilepticus. Epilepsia.

[5] Trinka E., Cock H., Hesdorffer D., Rossetti A.O., Scheffer I.E., Shinnar S., Shorvon S., Lowenstein D.H. (2015). A definition and classification of status epilepticus—Report of the ILAE Task Force on Classification of Status Epilepticus. Epilepsia.

[6] Blume W.T., Lüders H.O., Mizrahi E., Tassinari C., van Emde Boas W., Engel J. (2001). Glossary of descriptive terminology for ictal semiology: Report of the ILAE Task Force on Clasiffication and terminology. Epilepsia.

[7] Rosenow F., Hamer H., Knake S. (2007). The epidemiology of convulsive and nonconvulsive status epilepticus. Epilepsia.

[8] Skinner H.J., Dubon-Murcia S.A., Thompson A.R., Medina M.T., Edwards J.C., Nicholas J.S., Holden K.R. (2010). Adult convulsive status epilepticus in the developing country of Honduras. Seizure.

[9] Kortland L.M., Knake S., Rosenow F., Strzelczyk A. (2015). Cost of status epilepticus: A systematic review. Seizure.

[10] Dham B.S., Hunter K., Rincon F. (2014). The Epidemiology of status Epilepticus in the United States. Neurocrit Care.

[11] DeLorenzo R.J., Hauser W.A., Towne A.R., Boggs J.G., Pellock J.M., Penberthy L., Garnett L., Fortner C.A., Ko D. (1996). A prospective, population-based epidemiologic study of Status epilepticus in Richmond, Virginia. Neurology.

[12] Hesdorffer D.C., Logroscino G., Cascino G., Annegers J.F., Hauser W.A. (1998). Incidence of Status Epilepticus in Rochester, Minnesota, 1965–1984. Neurology.

[13] Waterhouse E.J., DeLorenzo R.J. (2001). Status epilepticus in the elderly patients: Epidemiology and treatment options. Drugs Aging.

[14] Hui A., Joynt G., Huan L.I., Wong K.S. (2003). Status epilepticus in Hong Kong Chinese: Aetiology, outcome and predictors of death and morbidity. Seizure.

[15] Li J.M., Chen L., Zhou B., Zhu Y., Zhou D. (2009). Convulsive status epilepticus in adults and adolescents of southwest China: Mortality, etiology and predictors of death. Epilepsy Behav..

[16] Nishiyama I., Ohtsuka Y., Tsuda T., Kobayashi K., Inoue H., Narahara K., Shiraga H., Kimura T., Ogawa M., Terasaki T. (2011). An epidemilogical study of children with status epilepticus in Okoyama, Japan: Incidence, etiologies, and outcomes. Epilepsy Res..

[17] Vignatelli L., Rinaldi R., Galeotti M., de Carolis P., D’Alessandro R. (2005). Epidemiology of Status Epilepticus in a rural area of northern Italy: A 2-year population based study. Eur. J. Neurol..

[18] Vignatelli L., Tonon C., D’Alessandro R. (2003). Incidence and short-term prognosis of status epilepticus in adults in Bologna, Italy. Epilepsia.

[19] Canouï-Poitrine F., Bastuji-Garin S., Alonso E., Darcel G., Verstichel P., Caillet P., Paillaud E. (2011). Risk and prognostic factors of status epilepticus in the elderly: A case-control study. Epilepsia.

[20] Coeytaux A., Jallon P., Galobardes B., Morabia A. (2000). Incidence of status epilepticus in french-speaking Switzerland (EPISTAR). Neurology.

[21] Chin R.F., Neville B.G., Peckham C., Bedford H., Wade A., Scott R.C. (2006). Incidence, cause, and short-term outcome of convulsive status epilepticus in chilhood: Prospective population based study. Lancet.

[22] Garzon E., Fernandes R.M., Sakamoto A.C. (2003). Analysis of clinical characteristics and risk factors for mortality in human status epilepticus. Seizure.

[23] Maldonado A., Ramos W., Pérez J., Huamán L.A., Gutiérrez E.L. (2010). Convulsive status epilepticus: Clinico-epidemiologic characteristics and risk factors in Peru. Neurologia.

[24] Amare A., Zenebe G., Hammack J., Davey G. (2008). Status epilepticus: Clinical presentation, cause, outcome and predictors of death in 119 Ethiopian patients. Epilepsia.

[25] Ozdilek B., Midi I., Agan K., Bingol C.A. (2013). Episodes of status epilepticus in young adults: Etiologic factors, subtypes, and outcomes. Epilepsy Behav..

[26] Urtecho J., Seifi A., Maltenfort M., Vibbert M., McBride W., Moussouttas M., Jallo J., Bell R., Rincon F. (2011). Incidence, risk factors, and impact of status epilepticus in sepsis in the United States. Crit. Care.

[27] Sadarangani M., Seaton C., Scott J.A.G., Ogutu B., Edwards T., Prins A., Gatakaa H., Idro R., Berkley J.A., Peshu N. (2008). Incidence and outcome of status epilepticus in Kenyan children: A cohort study. Lancet Neurol..

[28] Corey L.A., Pellock J.M., DeLorenzo R.J. (2004). Status epilepticus in a population-based Virginia twin sample. Epilepsia.

[29] DeLorenzo R.J., Pellock J.M., Towne A.R., Boggs J.G. (1995). Epidemiology of Status Epilepticus. J. Clin. Neurophysiol..

[30] Maytal J., Shinnar S., Moshe S.L., Alvarez L.A. (1989). Low morbidity and mortality of Status epilepticus in children. Pediatrics.

[31] Fountain N.B. (2000). Status epilepticus: Risk factors and complications. Epilepsia.

[32] Koubeissi M., Alshekhlee A. (2007). In-hospital mortality of generalized convulsive status epilepticus. A large US sample. Neurology.

[33] Tsai M.H., Chuang Y.C., Chang H.W., Chang W.N., Lai S.L., Huang C.R., Tsai N.W., Wang H.C., Lin Y.J., Lu C.H. (2009). Factors predictive of outcome in patients with de novo status epilepticus. QJM.

[34] Neligan A., Shorvon S.D. (2011). Prognostic factors, morbidity and mortality in tonic-clonic status epilepticus: A review. Epilepsy Res..

[35] Raspall-Chaure M., Chin R.F., Neville B.G., Scott R.C. (2006). Outcome of paediatric convulsive status epilepticus: A systematic review. Lancet Neurol..

[36] Waterhouse E.J., Vaughan J.K., Barnes T.Y., Boggs J.G., Towne A.R., Kopec-Garnett L., DeLorenzo R.J. (1998). Synergistic effect of status epilepticus and brain ischemic brain injury on mortality. Epilepsy Res..

[37] Shneker B.F., Fountain N.B. (2003). Assessement of acute morbidity and mortality in nonconvulsive status epilepticus. Neurology.

[38] Neligan A., Shorvon S.D. (2010). Frequency and prognosis of convulsive status epilepticus of different causes: A systematic review. Arch. Neurol..

[39] DeLorenzo R., Kirmani B., Deshpande L.S., Jakkampudi V., Towne A.R., Waterhouse E., Garnett L., Ramakrishnan V. (2009). Comparisons of mortality and clinical presentations of status epilepticus in private practice community and university hospital setting in Richmond, Virginia. Seizure.

[40] Waterhouse E.J., Garnett L.K., Towne A.R., Morton L.D., Barnes T., Ko D., DeLorenzo R.J. (1999). Prospective population-based study of intermittent and continuous convulsive status epilepticus in Richmond, Virginia. Epilepsia.

[41] Pujar S.S., Neville B.G., Scott R.C., Chin R.F. (2011). Death within 8 years after chilhood convulsive status epilepticus: A population-based study. Brain.

[42] Hesdorffer D.C., Logroscino G., Cascino G., Annegers J.F., Hauser W.A. (1998). Riks of unprovoked seizure after acute symptomatic seizure: Effect ot status epilepticus. Ann. Neurol..

[43] Ferro M.A., Chin R.F., Camfield C.S., Wiebe S., Levin S.D., Speechley K.N. (2014). Convulsive status epilepticus and health-related quality of life in children with epilepsy. Neurology.

[44] Claassen J., Lokin J.K., Fitzsimmons B.-F.M., Mendelsohn F.A., Mayer S.A. (2002). Predictor of functional disability and mortality after status epilepticus. Neurology.

[45] Penberthy L.T., Towne A., Garnett L.K., Perlin J.B., DeLorenzo R.J. (2005). Estimating the economic burden of status epilepticus to the health care system. Seizure.

[46] Strzelczyk A., Knake S., Oertel W.H., Rosenow F., Hamer H.M. (2013). Inpatient treatment costs of status epilepticus in adults in Germany. Seizure.

[47] WHO World Report on Ageing and Health. Proceedings of the 2014 IEEE Geoscience and Remote Sensing Symposium IGARSS.

[48] Morgenstern L.B., Smith M.A., Sánchez B.N., Brown D.L., Zahuranec D.B., Garcia N., Kerber K.A., Skolarus L.E., Meurer W.J., Burke J.F. (2013). Persistent ischemic stroke disparities despite delclining incidence in Mexican americans. Ann. Neurol..

[49] Barry E., Hauser W.A. (1994). Status epilepticus and antiepileptic medication levels. Neurology.

[50] Chin R., Neville B., Scott R.C. (2004). A systematic review of the epidemiology of status epilepticus. Eur. J. Neurol..

[51] World Health Organization (WHO) (2014). World Malaria Report.

[52] Greenwood B., Bojang K. (2005). Malaria. Lancet.

